# Multidetector dual-energy CT evaluation of combined partial anomalous pulmonary venous return and bronchial atresia

**DOI:** 10.1259/bjrcr.20150282

**Published:** 2016-01-19

**Authors:** Konstantinos Stefanidis, Charles Sayer, Ioannis Vlahos

**Affiliations:** Radiology Department, St George’s Hospital, London, UK

## Abstract

Partial anomalous venous return (PAPVR) and bronchial atresia (BA) represent rare congenital abnormalities of the lung. Missed diagnosis and misdiagnosis are very common in these patients. Although usually distinct entities, it appears that, in rare cases, they may co-exist owing to inter-related complex embryogenic development. We report a case of a 59-year-old male with both PAPVR and BA that were incidentally detected during a CT pulmonary angiogram and review the literature to suggest the pathogenetic developmental mechanism for this entity. This case demonstrates the utility of multidetector dual-energy CT in delineating the vascular and bronchial anatomy of this complex lung and vascular anomaly. Although uncommon, radiologists should be aware of PAPVR and BA and the coexistence of these two rare lung congenital abnormalities.

## Summary

Partial anomalous venous return (PAPVR) and bronchial atresia (BA) represent rare congenital abnormalities of the lung. Missed diagnosis and misdiagnosis are very common in these patients. Although usually distinct entities, it appears that, in rare cases, they may co-exist owing to inter-related complex embryogenic development. We report a case of a 59-year-old male with both PAPVR and BA that were incidentally detected during a CT pulmonary angiogram (CTPA) and review the literature to suggest the pathogenetic developmental mechanism for this entity. This case demonstrates the utility of multidetector dual-energy CT (DECT) in delineating the vascular and bronchial anatomy of this complex lung and vascular anomaly. Although uncommon, radiologists should be aware of PAPVR and BA and the coexistence of these two rare lung congenital abnormalities.

## Case presentation

A 59-year old male presented to the chest clinic with a history of intermittent left-sided pleuritic chest pain and progressive breathlessness on minimal exertion. His past medical history included repeated episodes of chest infection and a persistent right mid-zone opacity on serial chest radiographs. At the time, all other investigations were negative, including testing for mycobacterial disease and a reportedly normal flexible bronchoscopy at an outside institution. He was treated for many years with intermittent oral antibiotics and chest physiotherapy. He was a former smoker (20 pack-years) and also had a history of Type II diabetes mellitus, chronic obstructive airways disease and myocardial infarction with percutaneous coronary intervention.

## Investigations/imaging findings

**Figure 1.**
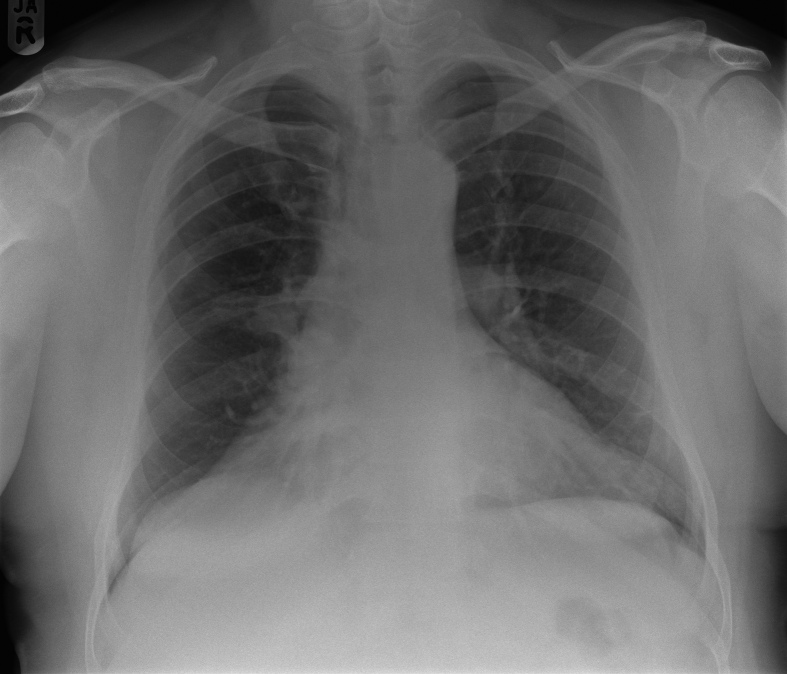
Pulmonary angiogram chest radiograph at presentation demonstrates right lung volume loss and a mid-zone opacity effacing the right cardiac border.

As part of his clinical work-up, a chest radiograph, CTPA, full pulmonary function tests and a transthoracic echocardiogram were requested. The echocardiogram was normal, with preserved left ventricle function and no evidence of raised pulmonary artery pressures. Pulmonary function tests revealed a mild obstructive picture (forced vital capacity 2.1 l, forced expiratory volume in 1 s 75% predicted). A posteroanterior chest radiograph demonstrated right lung volume loss and a right mid-zone opacity (Figure 1), appearances that had remained unchanged for many years. A dual energy (DE)-CTPA was performed as per our institution’s protocol for the investigation of suspected pulmonary embolism (PE; SOMATOM Definition Flash; Siemens Medical Solutions, Forchheim, Germany) as follows: full inspiration; 100 ml iohexol (300 mgI ml^–1^) administered at 5 ml s^–1^ with a 40 ml saline chaser administered at 5 ml s^–1^; scan delay determined by bolus tracking of a region of interest over the central pulmonary artery (trigger threshold 100 HU). Scan parameters of 100 kVp and tin-filtered (Sn) 140 kVp at effective 150 and 128 mAs, respectively; 128 × 0.6 mm collimation; 0.28  s rotation time; 0.8 pitch. Image reconstruction at 1 mm thickness at 0.8 mm intervals by using a soft convolution algorithm (D30) at 100  and Sn 140 kVp with a 0.8 mm interval weighted-average dataset (40% : 60% weighting from the Sn 140 and 100 kVp images, respectively).

**Figure 2.**
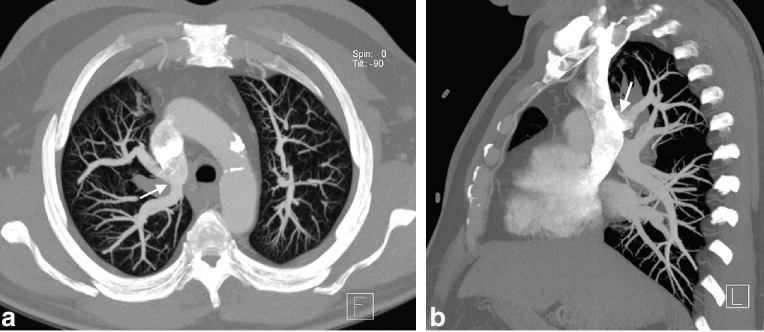
(a, b) Axial and sagittal 25-mm maximum-intensity projection contrast-enhanced images show the right upper lobe pulmonary veins draining into the posterior aspect of the superior vena cava (arrows).

**Figure 3.**
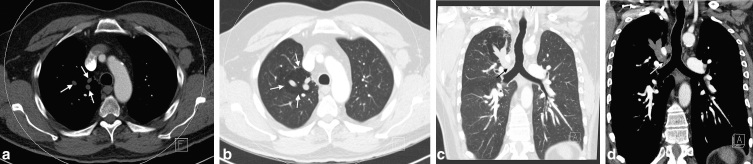
Axial imaging demonstrates mucoid-impacted airways (a) and air trapping (b) (arrows). Coronal reconstruction shows absence of the right apical segmental bronchus, with no communication between the right main bronchus and the mucoid-impacted airways (c, d) (arrows).

**Figure 4.**
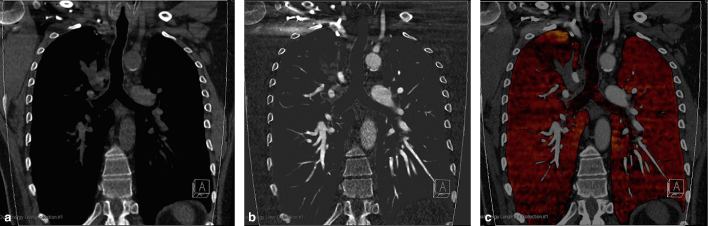
(a) Virtual non-contrast and (b) iodine selective images demonstrate no calcification or enhancement within the bronchocele in the RUL. (c) Pulmonary blood volume map demonstrates a wedge-shaped area of reduced perfusion in the RUL corresponding to the region of air trapping secondary to physiological shunting away from the redundant pulmonary segment. RUL, right upper lobe.

**Figure 5.**
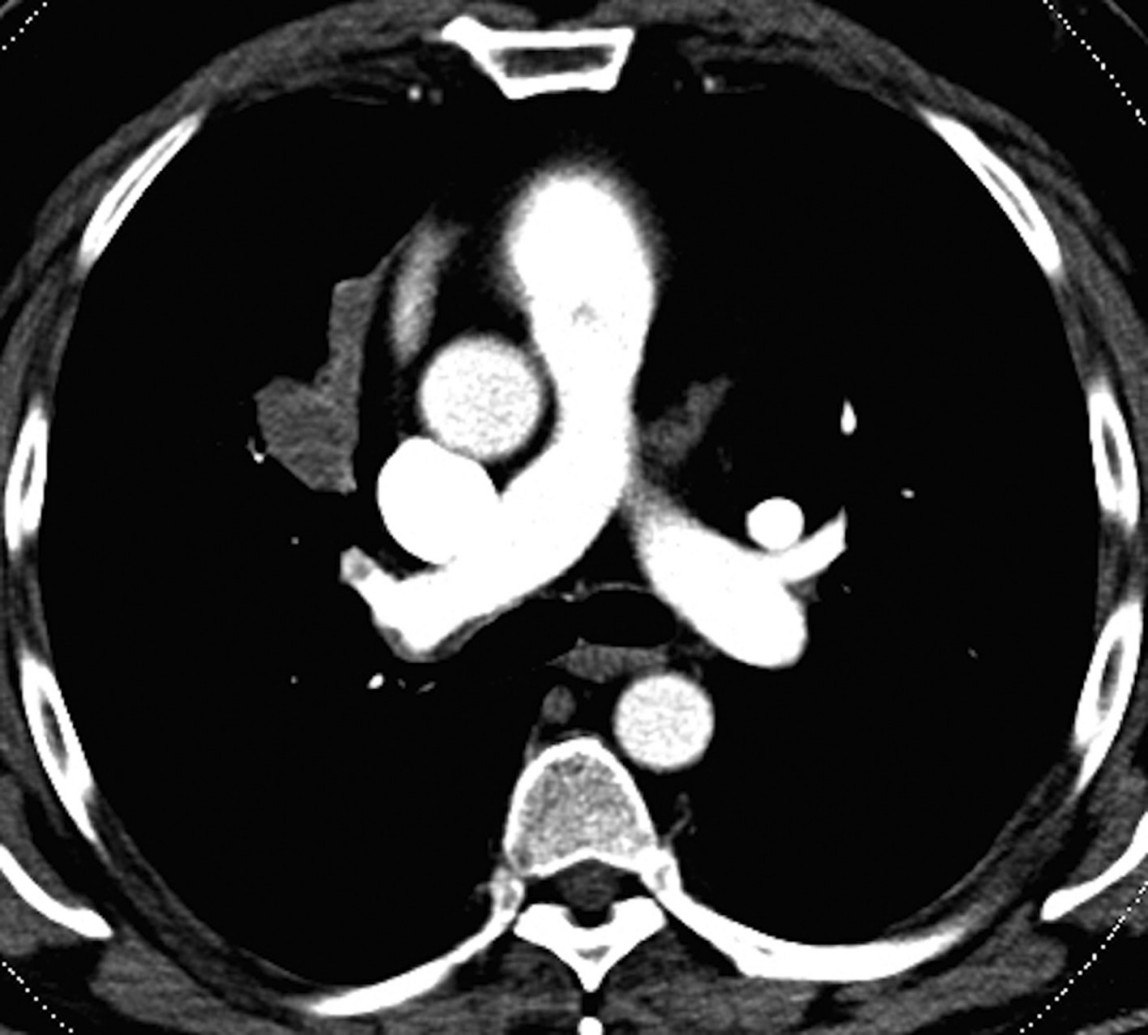
Axial imaging shows a small filling defect in the right segmental pulmonary artery and the long-standing soft-tissue lesion in the inferomedial right upper lobe.

The CTPA showed a right upper lobe (RUL) PAPVR with anomalous pulmonary venous connection between the RUL pulmonary veins and the superior vena cava (SVC; Figure 2a,b). In addition, BA was identified in the RUL with mucoid-impacted airways extending peripherally into the RUL and proximally tapered to a point occlusion (Figure 3a). The lung parenchyma distal to the BA demonstrated air trapping (Figure 3b) and no communication was demonstrated between the right apical segmental bronchus and the RUL bronchus (Figure 3c,d). DE image reconstruction and postprocessing confirmed the diagnosis of bronchocele with absence of an intrabronchial enhancing lesion and further demonstrated the reduced pulmonary perfusion (Figure 4a–c). In addition, an inferomedial RUL bronchial low-density opacity suggestive of an additional area of mucoid impaction was identified in the right paracardial region, likely accounting for the chronic chest radiograph abnormality (Figure 5). With the exception of a small, isolated, segmental right middle lobe artery embolism that subsequently resolved, the rest of the abnormalities were stable 1 year later.

## Discussion

CT imaging is integral in modern medicine, providing excellent anatomical detail and diagnostically valuable information for a wide spectrum of congenital abnormalities and pathological conditions. The use of multidetector isotropic, high-spatial resolution datasets with multiplanar reformation (MPR) and three-dimensional (3D) reconstructions has assumed a greater role in the non-invasive evaluation of congenital lung anomalies. Additionally, the use of DECT with its ability to evaluate enhancement, and potentially ventilation, allows further characterization of a spectrum of lung and vascular abnormalities.^1,2^

PAPVR and BA are uncommon congenital anomalies, usually found incidentally during diagnostic studies or a surgical procedure performed for other reasons. PAPVR is characterized by an anomalous pulmonary vein, more typically in the left upper lobe that connects to the left innominate vein, coursing cephalad along the prevascular space. Right-sided PAPVR is less common, draining usually from the RUL to the SVC or azygos vein, or directly to the right atrium.^3^ This may occur as an isolated anomaly or in association with an atrial septal defect (typically of the sinus venosus variant), particularly in patients with RUL PAPVR.^4^ Practically, it results in an extracardiac left-to-right shunt, as oxygenated pulmonary venous blood flows directly into the right side of the heart or systemic veins.

BA is another uncommon developmental abnormality first described by Ramsay and Byron in 1953.^5^ BA is characterized by proximal bronchus atresia or obstruction, with normal development of the distal airway. In this rare anomaly, the apicoposterior segment of the left upper lobe bronchus is involved most frequently, followed by the segmental bronchi of the right upper, middle and right lower lobes.^6^ This condition is associated with mucoid-impacted airways distal to the atretic segment and hyperinflation or emhpysema of the involved segment of the lung that is ventilated by collateral airflow through the pores of Kohn and canals of Lambert.

Although the coexistence of these two different congenital abnormalities has been described in only three cases in the literature, it appears that these structural entities may be interlinked during embryogenic development.

The embryological development of the pulmonary veins is controversial. Previously, there was no consensus as to whether they originate as buds from the left atrium and then connect to the lung plexus or if they develop from a single vein in the dorsal mesocardium and become incorporated into the left atrium.^7^ Nonetheless, it is generally agreed that the pulmonary vein canalizes at approximately 6 weeks of gestation before incorporation into the left atrium.^8^ Modern molecular staining techniques show that it represents a new structure that is secondarily incorporated into the developing atrium and does not originate from the sinus venosus or primary heart tube as previously thought.^9^ Therefore, the incorporation of the separate pulmonary veins into the left atrium occurs after 6 weeks of gestation, following the regression of pulmonary venous connections to systemic veins by day 40.^10^ Failure of this regression in all these connections can result in persistent drainage to the systemic circulation, the SVC in our case.

As the development of the airways precedes the development of the pulmonary veins, Meng et al^11^ hypothesized that an abnormal development of the RUL pulmonary vein at this stage could compromise the already developed RUL bronchus, resulting in an atretic segment and the subsequent appearance of “congenital” BA. As a consequence, normal branching distal to the atresia is maintained, as demonstrated in our case. In this complex cascade of embryological abnormalities, abnormal lung lobulation seems to be related to the sequela of the combined lung and vascular abnormalities.^12^ Finally, extrinsic compression and formation of an atretic bronchus might predispose to the development of bronchopulmonary sequestration.^13,14^

To our knowledge, the combination of these rare abnormalities is extremely unusual, having been reported in only three cases in the literature. Siddiqui et al^15^ reported a case of anomalous superior pulmonary venous return into the left brachiocephalic vein with coexistant BA in an asymptomatic patient with an abnormal chest X-ray. Kavakli et al^16^ described a case with PAPVR to the azygos vein with an absent RUL posterior segmental bronchus in which thoracoscopic right upper lobectomy was performed. In a more recent case report, an infected cystic lesion was detected as a complication in a young patient with congenital BA and PAPVR in the RUL.^17^

Not only in our case, but also in the case of Okada et al,^17^ drainage of the aberrant RUL pulmonary vein has a rather sagittal orientation when entering the posterior SVC compared with the more common coronal orientation with lateral insertion into the SVC in isolated RUL PAPVR. We hypothesize that the orientation of the anomalous vein may be contributing to concomitant RUL BA.

Right-sided PAPVR is usually associated with a sinus venosus atrial septal defect (SVASD), which may have significant clinical importance depending on the degree of left-to-right shunt. However, in our case, no SVASD or other congenital heart disease was shown. BA may be an asymptomatic incidental finding or associated with recurrent infections, as in our case.

Radiographical findings of PAPVR and BA depend on the location, the presence of associated complications, the configuration of anomalous drainage and the degree of left-to-right shunt associated with the PAPVR (or a coexistant SVASD). Chest radiography is commonly the initial imaging examination. This may be normal or show increased pulmonary artery size or branching opacities. CT scan is more sensitive for the detection of related abnormalities such as cystic mixed lesions, emphysematous changes, fluid collection, nodules or consolidation of various sizes secondary to chronic or recurrent pulmonary infections.^6^ Multidetector CT scan with postprocessing methods, including MPR and 3D volume rendering, is the modality of choice in the detection of vascular and bronchial abnormalities.^18,19^ Reconstruction of all vessels in any orientation and depiction of a lack of communication between the origin of the proximally obstructed bronchus and the dilated branching plugged airways is possible.^19^ The additional advantages of DECT may be the ability to exclude any enhancing endobronchial abnormality simulating BA and better define the pulmonary and vascular perfusion in the affected segment.

The introduction of DECT in the last decade has provided additional information in thoracic imaging work-up of PE; characterization of lung neoplasms and lymph nodes; and visualization of lung perfusion, ventilation and myocardial perfusion.^20–23^ While the existing literature suggests that there is no increase in radiation exposure in DECT, the creation of virtual unenhanced images from a contrast-enhanced DECT dataset can reduce the overall radiation burden by replacing an unenhanced scan.^24^

The combination of BA with PAPVR is an extremely uncommon disorder. Although uncommon, congenital lung and vascular anomalies such as PAPVR and BA have to be recognized by radiologists. The recognition of developmental anomalies is important because they frequently mimic other acquired abnormalities. Indeed, in this case, the diagnosis was occult for many years, leading to ineffective treatment for ongoing patient symptomatology and morbidity. It is hoped that this article will contribute to understanding the usefulness of modern imaging in the recognition of these rare congenital abnormalities and provide additional information to referring surgeons for preoperative planning.

## Learning points

1. Although uncommon, congenital lung and vascular anomalies, such as PAPVR and BA, have to be recognized for effective treatment and possible preoperative planning.
2. The combination of these two congenital abnormalities is extremely rare and may be related to the same process during embryogenesis.
3. PAPVR and BA are uncommon congenital anomalies.
4. Multidetector DECT is an essential, non-invasive tool in the recognition and delineation of the anatomy and further characterization of these rare congenital anomalies.
